# Preharvest long-term exposure to UV-B radiation promotes fruit ripening and modifies stage-specific anthocyanin metabolism in highbush blueberry

**DOI:** 10.1038/s41438-021-00503-4

**Published:** 2021-04-01

**Authors:** Taishan Li, Hisayo Yamane, Ryutaro Tao

**Affiliations:** Graduate School of Agriculture, Kyoto University, Kyoto, 606-8502 Japan

**Keywords:** Light responses, Secondary metabolism

## Abstract

Ultraviolet-B (UV-B) light (280–315 nm) is an important environmental signal that regulates plant development and photomorphogenesis, while also affecting the flavonoid pathway, including anthocyanin biosynthesis. Regarding the effects of UV-B radiation on fruits, the effects of a short-term or postharvest irradiation on fruit quality have been well-documented, but the effects of a long-term preharvest UV-B irradiation on fruit growth and coloration remain unclear. Thus, in this study, we investigated the effects of a long-term treatment involving an environmentally relevant UV-B dose on highbush blueberry (*Vaccinium corymbosum*) fruit. The preharvest UV-B treatment quickly promoted fruit growth and sugar accumulation, which is not commonly observed in other fruit tree species. The UV-B exposure also accelerated fruit ripening and coloration. The dual-luciferase assay proved that in blueberries, expression of *VcUFGT* encoding anthocyanin biosynthesis key enzyme, is positively and negatively regulated by *VcMYBA1* and *VcMYBC2*, respectively. Throughout the fruit development stage, the UV-B treatment up-regulated *VcMYBPA1* expression, which increased *VcUFGT* expression via *VcMYBA1*. In the green fruit stage, the UV-B treatment increased *HY5* encoding UV receptor, which up-regulates *VcMYBPA1* and down-regulates *VcMYBC2*, thereby promotes the accumulation of anthocyanins. On the other hand, excessive anthocyanin synthesis was inhibited by increased *VcMYBC2* levels in mature fruits when exposed to UV-B light through *HY5*-independent pathway. In conclusion, anthocyanin-related MYB activators and repressor may coordinately balance the accumulation of anthocyanins in blueberry fruits, with UV-B treatments possibly influencing their effects in a stage-specific manner. The potential utility of preharvest UV-B treatments for improving blueberry fruit quality is discussed herein.

## Introduction

Blueberry (*Vaccinium* spp.) is known for its relatively high anthocyanin contents that can decrease the risk of developing coronary artery disease or gut inflammation, while also positively affecting the nervous tissue in the brain^1,2^. In northeastern China, blueberry fruits are increasingly being produced in greenhouses because forcing cultivation in greenhouses enables early harvesting, which usually leads to increased profits for farmers^3^. However, one of the notable differences between greenhouse cultivation and field cultivation is the lack of ultraviolet-B (UV-B) light (280–315 nm) in glass greenhouses and greenhouses with plastic films that absorb UV-B radiation^4,5^. Although UV-B radiation accounts for only a small proportion of the radiation reaching the Earth’s surface, it can substantially affect plant metabolic responses to oxidative stress as well as plant growth and development^6^. Exposing plants to UV-B light can induce the biosynthesis of flavonoids, especially anthocyanins. Previous research on grapes (*Vitis vinifera*)^7^, apples (*Malus* × *domestica*)^8^, and strawberries (*Fragaria* spp.)^9^ as well as analyses of postharvest and short-term preharvest treatments of blueberries^10,11^ revealed that targeted UV-B irradiation can enhance the production of phenylpropanoid substances in highly nutritious fruits and vegetables. However, the effects of UV-B radiation on the fruit growth and development of greenhouse-cultivated fruit crops remain relatively unknown.

In plants, anthocyanin biosynthesis is mediated by the anthocyanin pathway involving structural genes and regulatory transcription factors (TFs) contributing to the production of diverse anthocyanin components. This pathway has been thoroughly characterized^12^, with the genes encoding the following anthocyanin biosynthetic enzymes having been identified: chalcone synthase (CHS), flavonoid 3′-hydroxylase (F3′H), flavonoid 3′,5′-hydroxylase (F3′5′H), dihydroflavonol reductase (DFR), anthocyanidin synthase (ANS), uridine diphosphate (UDP)-glucose:flavonoid-O-glycosyl-transferase (UFGT), flavonol synthase (FLS), leucoanthocyanidin reductase (LAR), and anthocyanidin reductase (ANR). Recent research on blueberry has mainly focused on the gene encoding UGFT, with its substantial increase in transcription in mature fruits, suggesting the *UFGT* is important for the rapid accumulation of anthocyanins in maturing fruit^13–15^. The MBW complex, which comprises the R2R3-MYB TF, basic helix-loop-helix (*bHLH*) TF, and a WD repeat (WDR)-containing protein, is the core regulator that directly controls anthocyanin production in plants by promoting the expression of anthocyanin biosynthesis-related structural genes^16^. The R2R3-MYB TF genes from subgroups (SGs) 5 and 6 include anthocyanin-related MYB activator genes, such as *AtMYB75* (PAP1), *AtMYB90* (PAP2), *AtMYB113*, and *AtMYB114* in *Arabidopsis thaliana*, *VvMYBA1* and *VvMYBA2* in grapevine, *MdMYB10* and *MdMYB110a* in apple, and *PpMYB10.1* in peach, as well as proanthocyanidin (PA)-related MYB activators, such as *AtMYB123* and *AtMYB5* in *A. thaliana* and *VvMYBPA1*, *VvMYBPA2*, *VvMYB5a*, and *VvMYBPAR* in grapevine^17^. In addition to the MYB activators, MYB inhibitors have important regulatory functions influencing flavonoid metabolism, especially anthocyanin biosynthesis. In grapevine, four R2R3-MYB TFs with the C2 repressor motif may be key negative regulators of the synthesis of small phenolic compounds that precisely control flavonoid levels and balance the inductive effects of transcriptional activators^18^. In peach, the expression of *PpMYB18*, which is a homolog of *VvMYBC2-L1*, can negatively regulate the accumulation of anthocyanins and PAs^19^. Additionally, PpMYB18 and the MYB activator PpMYB10 help form a negative feedback loop that prevents the excessive accumulation of anthocyanins and PAs^19^. In blueberry, two MYB activator genes, *VcMYBA1* from SG6 and *VcMYBPA* from SG5, and one MYB repressor gene, *VcMYBC2*, have been identified^13,20,21^, however, the effects of the encoded TFs on *VcUFGT* has not been investigated. Moreover, MYB repressor function has yet to be verified.

In plants, UV-B signal transduction is mediated by UV-B-specific photoreceptors (e.g., UVR8) that induce the transcription of signal transduction genes, such as *CONSTITUTIVELY PHOTOMORPHOGENIC 1* (*COP1*) and *ELONGATED HYPOCOTYL 5* (*HY5*), to regulate light-dependent responses and gene expression^22^. Under UV-B light, the UVR8 photoreceptor interacts with COP1, which leads to an increase in HY5 expression through a positive regulatory mechanism. In darkness, HY5 is degraded by COP1. In apple, the greater accumulation of anthocyanins in UV-B-treated fruit peels than in control peels not exposed to UV-B radiation is due to the up-regulated expression of *MdHY5* and *MdMYB10*^8^. However, the UV-B-responsive molecular pathways underlying UV-B-induced fruit maturation and anthocyanin accumulation in blueberry has not been elucidated.

In this study, we evaluated the effects of a long-term environmentally based UV-B treatment on blueberry cultivated in a greenhouse. The objective of this study was to clarify the effects of UV-B light on blueberry fruit development and maturation. We focused specifically on the molecular regulatory network underlying UV-B-triggered anthocyanin metabolism, which revealed the stage-specific UV-B-responsive fruit coloration mechanism and a novel regulatory network fine-tuning the anthocyanin accumulation in blueberry fruits.

## Materials and methods

### Plant materials and UV-B treatments

**Fig. 1:**
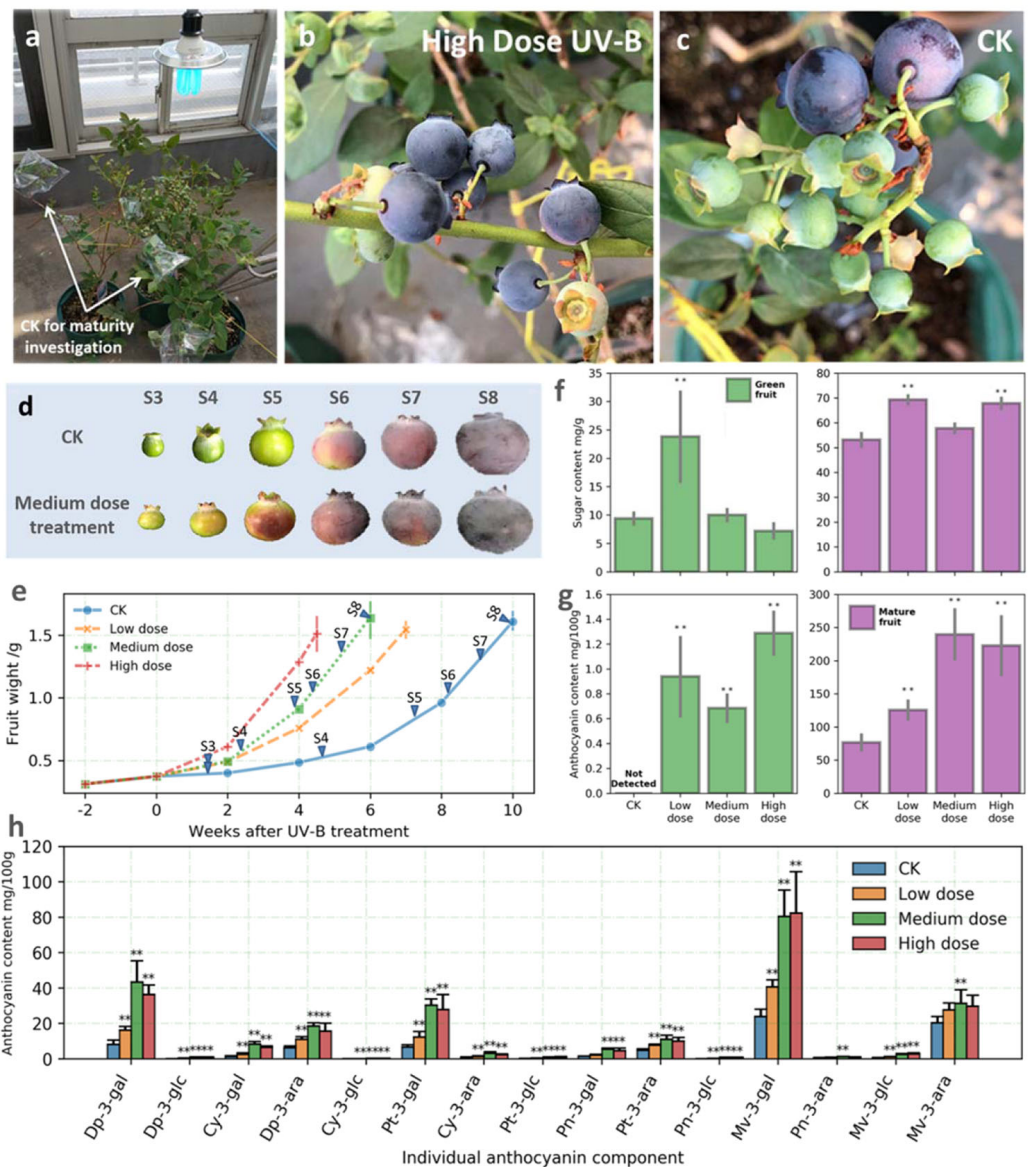
Effects of UV-B on blueberry fruit development, maturation, and anthocyanin accumulation. **a** UV-B treatment set-up in the greenhouse. **b** Effect of a high UV-B dose on fruit ripening after 2-week treatment. **c** Effect of the control (CK) conditions on fruit ripening after 2-week treatment. **d** Effects of UV-B on fruit anthocyanin accumulation, the stage label corresponded to the description in Zifkin et al. ^13^. **e** Averaged fruit size development of 15 fruits in each treatment, markers with stage label corresponded to the fruits in (**d**). **f** Sugar (upper panel) and anthocyanin (lower panel) accumulation of green (left panel) and mature (right panel) fruits. **g** anthocyanin components of mature fruits. **f**, **g** (mean±s.e., *n* = 3). ‘*‘(*P* < 0.05) and ‘**‘(*P* < 0.01) indicates the significant difference between treatment and CK

Two- to three-year-old pot-grown shrubs of southern highbush blueberry (*V. corymbosum*) interspecific hybrid ‘O’Neal’ were used in this study. They were grown in a greenhouse located at the Kyoto Farmstead of the Experimental Farm, Kyoto University, Japan. The temperature in the greenhouse was maintained at 25 °C during the day and 20 °C at night with an air conditioner. The preharvest UV-B irradiation experiment was conducted during the 2018 growing season. A UV-B-absorbing film (Achilles nonkiri toshimasen, UV-cut type, 0.075 mm × 300 cm) that can absorb 99% UV-B light, was used to cover fruits to obtain the control fruits (CK) to ensure that the treatments were unaffected by solar UV rays. The plant positions were adjusted so that all treated fruits were exposed to the same irradiation intensity. At 6–7 weeks after blooming, we exposed ‘O’Neal’ plants to various UV-B doses. At least 5 shrubs were used for each treatment. More than 10 branches bearing at least 3 fruit clusters were applied to each treatment. UV-B treated group were supplied with ultraviolet via UV-B lamps (PWFD24UB1PB; Panasonic, Osaka, Japan), and fruits in CK were covered with UV-B absorbing films. The seasonal doses of biologically effective UV-B radiation in this experiment were determined based on a previous study^23^. The biologically effective UV-B radiation for the UV-B lamps was calculated with the generalized curve described by Caldwell^24^. The UV-B treatments involved an exposure to the following three biologically effective doses for 7 h per day: 1) a low-dose treatment representing the UV dose of an average winter day (0.07 W/m^2^); 2) a medium-dose treatment representing the UV dose of an average summer day (0.14 W/m^2^); and 3) a high-dose treatment with 30% more UV irradiation than an average summer day (0.19 W/m^2^). Fruit clusters with the UV-B-absorbing film during the UV treatments served as the control group (CK) (Fig. 1a). Fruits at 1 week after starting the UV-B treatment (55 days after blooming) were harvested and used for subsequent analysis as “green fruits” samples, whereas fruits at S8-stage defined by Zifkin et al.^13^ were harvested and used as “mature fruits” samples. Fruits were collected 2 h after the UV treatment on each sampling day. The pericarp was separated from the flesh, immediately frozen in liquid nitrogen, and stored at −80 °C until analyzed.

### Expression analysis of UV receptor, anthocyanin-related MYB, and structural genes in each fruit stage

Total RNA was extracted from the pericarp of the collected fruit samples using the CTAB–KAc^25^and then reverse transcribed with the ReverTra Ace^®^ qPCR RT Master Mix with gDNA Remover (Toyobo, Osaka, Japan). A search for sequence similarities revealed genes encoding putative UV receptors, anthocyanin- and proanthocyanidin-related MYB proteins, as well as structural genes in the blueberry genome^14^. Sequence similarities were assessed based on the Clustal V multiple sequence alignments produced with MEGA7. A phylogenetic analysis of *V. corymbosum*, *V. vinifera*, *Prunus persica*, *M. domestica*, and *A. thaliana* MYB proteins was completed according to the maximum likelihood method (http://www.phylogeny.fr)^26^. Specifically, the MUSCLE program was used to align protein sequences, after which maximum likelihood phylogenetic trees were constructed, with 500 bootstrap replicates. Quantitative reverse transcription PCR (qPCR) analyses were conducted with the LightCycler 480 system (Hoffmann-La Roche, Basel, Switzerland) and the THUNDERBIRD^®^ SYBR qPCR Mix (Toyobo). The qPCR conditions were as follows: 95 °C for 5 min; 40 cycles of 95 °C for 5 s and 58 °C for 1 min. The *VcGAPDH* gene was used as the reference control as previously described^13^. The gene-specific primers are listed in Table S1.

### Analyses of anthocyanin compositions and sugar contents

Anthocyanins were extracted and analyzed as described by Irizumi et al^27^. Briefly, frozen pericarp samples were ground to a powder with the Multi-beads shocker (Yasui Kikai, Osaka, Japan) and 0.2 g fine powder was resuspended in 5 mL 0.1% (v/v) HCl–methanol and incubated at 10 °C for 40 min. The solution was then centrifuged at 13,000 × *g* at 4 °C for 15 min. The supernatant was analyzed with a high-performance liquid chromatography (HPLC)-photodiode array system. The Wakopak Wakosil-II 5C18 RS column (4.6 mm × 150 mm) was used for the chromatographic analysis. The mobile phase was solvent A (1.5% phosphoric acid aqueous solution) and solvent B (1.5% phosphoric acid, 20% acetic acid, and 25% acetonitrile aqueous solution). The flow rate was 0.75 mL/min, with a column temperature of 40 °C and a detection wavelength of 520 nm. The gradient elution was completed with an increasing solvent B concentration from 15% to 55% in 80 min. The relative anthocyanin contents were calculated as milligrams of cyanidin-3-glucoside chloride equivalents per 100 g fresh weight. Each sample was analyzed with three replicates.

To quantify the sugar content, 0.5 g ground pericarp powder was resuspended in 5 mL ultra-pure water for a 40-min ultrasonic extraction, which was followed by a centrifugation at 12,000 × *g* for 10 min. The supernatant was analyzed by HPLC with a refractive index detector and a Shim-pack CLC column (4.6 mm × 250 mm) (Shimadzu, Kyoto, Japan). The mobile phase was 87.5% acetonitrile and the gradient elution was completed at a flow rate of 1.2 mL/min, with a column temperature of 40 °C. The sample injection volume was 10 μL. The glucose, fructose, and sucrose contents were determined and total sugar content was calculated as the sum of these three kinds of sugars. Each sample was analyzed with three replicates.

### Dual-luciferase assay

Dual-luciferase assays were performed using tobacco (*Nicotiana benthamiana*) leaves as previously described^28,29^. The *VcUFGT*, *VcMYBPA1*, *VcMYBA1*, and *VcMYBC2* promoter sequences identified from the blueberry genome sequence were examined for cis-regulatory elements with the plant cis-acting regulatory DNA elements database (http://www.dna.affrc.go.jp/PLACE/index.html)^30^. *MdbHLH3* has been reported as an interacted bHLH protein with *VcMYBA1*^20^, thus it was cloned from apple leaves and used as the MYB-binding bHLH protein in this research. The full-length coding sequences of the *VcHY5*, *VcMYBPA1*, *VcMYBA1*, *VcMYBC2* and *MdbHLH* were separately cloned from ‘O’Neal’ and inserted into the multiple cloning sites of the pGreenII 0029 62-SK vector, whereas the *VcUFGT*, *VcMYBPA1*, *VcMYBA1*, and *VcMYBC2* promoter sequences were inserted into the pGreenII 0800-LUC vector to drive the expression of the luciferase gene. The primers used to amplify the full-length coding sequences and promoter sequences are listed in Table S2. *Agrobacterium tumefaciens* GV3101 cells were separately transformed with the recombinant plasmids via electroporation. Tobacco leaves were then infiltrated with the transformed *A. tumefaciens* cells, after which the transient expression and LUC (firefly luciferase) and REN (*Renilla* luciferase) enzyme activities were analyzed. Specifically, at 3 days after the infiltration, LUC and REN activities were examined with the Dual-Luciferase Reporter Assay system (Promega, Madison, WI, USA) and the Modulus luminometer (Promega). The analysis was completed with three independent experiments, with four biological replicates per assay.

### Statistical analysis

Experiments were conducted according to a completely randomized design and treatments comprised five replicates, and all samples were analyzed in triplicate. Data were analyzed with SPSS 19.0 (SPSS Inc., USA). The *t*-test was used to compare the differences between the treatment and control groups, with * and ** indicating significant differences at *P* < 0.05 and *P* < 0.01, respectively.

## Results

### A preharvest UV-B treatment promoted blueberry fruit development and maturation

An exposure to UV-B light significantly enhanced blueberry fruit growth and development, leading to accelerated maturation and ripening (Fig. 1). The fruit harvest dates (i.e., when 50% of the fruits were S8-stage^13^) for the plants exposed to high, medium, or low UV-B doses were about 5.5, 4, and 3 weeks earlier than that of the CK plants, respectively (Fig. 1e). Clear differences were detected in the CK and high UV-B dose-treated fruit clusters on the same experimental shrub at 2 weeks after the UV-B treatment (Fig. 1b, c). The high-dose UV-B treatment resulted in faster and more consistent fruit maturation. In contrast, the CK fruit clusters matured later and more unevenly, with some fruits remaining in the young-green stage. These observations reflected the potential utility of UV-B irradiation for regulating the fruit maturation period and decreasing the cost of artificial harvesting. Regarding the final fruit weight, the low and medium UV-B doses had no effect, whereas the high dose decreased the average fruit weight compared with the CK fruit weight; however, this difference was not significant (Fig. 1e). The accumulation of sugars (glucose, fructose, and sucrose) significantly increased in green fruits only in response to the low UV-B dose, whereas it increased in the mature fruits (relative to that in the CK fruits) following the treatments with low and high UV-B doses (Fig. 1f).

### A preharvest UV-B treatment induced blueberry fruit coloration and anthocyanin accumulation

Anthocyanins were undetectable in the CK pericarp at the green fruit stage, but the exposure to UV-B light induced the fruit skin coloration and anthocyanin accumulation (Fig. 1d, g and Fig. S1A). Compared with the CK fruit samples, the mature fruit anthocyanin contents increased by 167% and 148% after the treatments with medium (0.14 W/m^2^) and high (0.19 W/m^2^) UV-B doses, respectively (Fig. 1g). Only eight different anthocyanin components were identified in UV-B-treated green fruits, whereas 11 anthocyanin components were detected in the initial maturity fruit peels (Fig. S1). The abundance of each anthocyanin component in mature fruits was determined (Fig. 1h). Delphinidin-3-galactoside (Dp-3-gal), petunidin-3-galactoside (Pt-3-gal), malvidin-3-galactoside (Mv-3-gal), and malvidin-3-arabinopyranoside (Mv-3-ara) were identified as the main anthocyanin components in the ‘O’Neal’ fruit. Among various anthocyanin forms, Mv-3-gal was predominant in ‘O’Neal’ fruit, which is in consistent with previous study^21^. After the UV-B treatment, the content of three of these components, Dp-3-gal, Pt-3-gal, and Mv-3-gal, increased significantly, and the change in these three components accounted for more than 80% of the total anthocyanin increase induced by the UV-B radiation.

### Expression analysis of genes related to the anthocyanin biosynthesis pathway and the UV-B signal transduction pathway

**Fig. 2:**
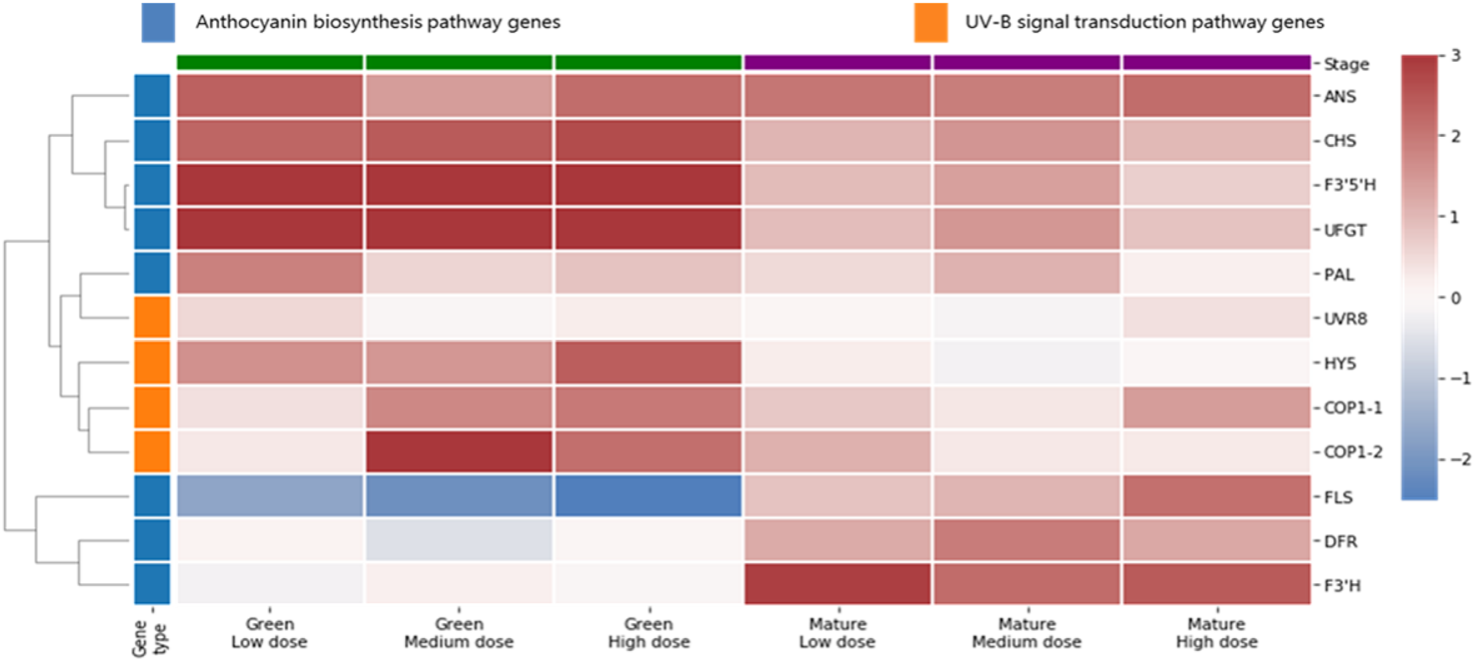
Expression analysis of UV-B signal transduction and anthocyanin biosynthesis pathway genes. The values in the heat map represent the log_2_-fold change (expression levels in treated fruits/CK fruits; *n* = 4)

The expression-level fold-changes between the CK and UV-B-treated fruit samples at the green and mature fruit stages were determined for the structural genes in the anthocyanin and flavonol biosynthesis pathway (VcCHS, VcF3’H, VcF3’5′H, VcDFR, VcANS, VcUFGT, and VcFLS) and in the UV-B response pathway (VcUVR8, VcCOP1-1, VcCOP1-2, and VcHY5) (Fig. 2). The structural genes were divided into three groups according to their expression-level changes after the UV-B treatment. The expression of four genes (VcUFGT, VcF3’5′H, VcCHS, and VcANS) was significantly (*P* < 0.01) enhanced by the UV-B light at the green and mature fruit stages, whereas the expression of two genes (VcDFR and VcF3’H) was up-regulated by the UV-B treatment only during the mature fruit stage. In response to the UV-B treatment, VcFLS expression decreased significantly (*P* < 0.01) at the green fruit stage, but increased significantly (*P* < 0.01) in mature fruits.

Regarding the UV-B-responsive genes, the expression of UVR8 in developing fruit was unaffected by UV-B light, whereas VcCOP1-1 and VcCOP1-2 expression exhibited dose-dependent changes after the UV-B treatments. Medium and high UV-B doses significantly (*P* < 0.01) up-regulated the expression of these two COP1 genes in the green fruit stage. At the mature fruit stage, the expression of these genes was generally up-regulated by all three UV-B doses. Although the UV-B irradiation up-regulated VcHY5 expression at the green fruit stage, it had no obvious effects on the expression of this gene in mature fruits.

### Identification and characterization of activator- and repressor-type R2R3-MYB TFs in blueberry and their expression due to UV-B treatments

**Fig. 3:**
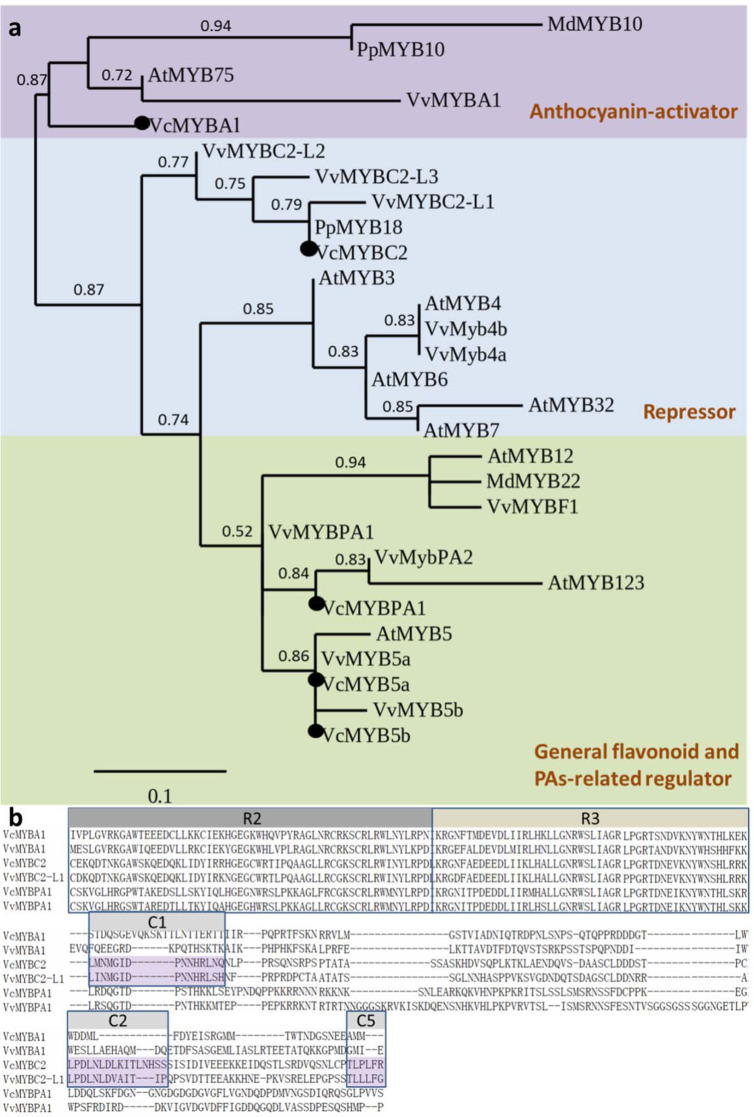
Identification of anthocyanin biosynthesis-related MYB proteins in blueberry. **a** Phylogenetic tree based on the amino acid sequences of R2R3‐MYB transcription factors in blueberry and other species. **b** Amino acid sequence alignment of VcMYBA1, VcMYBPA1, *VcMYBC2*, and other known flavonoid-related MYB activators in plants. The R2 and R3 domains are indicated with gray boxes. The conserved C-terminal motifs (C1, C2, and C5) in MYBC2-type proteins are indicated

Five blueberry genes (*VcMYBA*, *VcMYBC2*, *VcMYBPA1*, *VcMYB5a*, and *VcMYB5b*) encoding MYB TFs involved in anthocyanin synthesis were cloned from ‘O’Neal’ and revealed in our phylogenetic analysis (Fig. 3a). The *VcMYBA1* and *VcMYBPA1* genes were originally described by Plunkett^20^ and Zifkin^13^. However, *VcMYBC2*, *VcMYB5a*, and *VcMYB5b* were identified for the first time in this study based on their homology to *VvMYBC2-L1*, *VvMYB5a*, and *VvMYB5b*, respectively, in the grape genome. The *VcMYBC2* gene comprises 1,236 bp predicted to encode an R2R3-MYB TF with 233 amino acid residues. It is phylogenetically related to *PpMYB18*^19^ and *VvMYBC2-L1*, which negatively regulate anthocyanin accumulation in peach^19^ and grape^18^, respectively. Three conserved repressor motifs, C1, C2, and C5, were identified in *VcMYBC2* (Fig. 3b), suggesting this gene may encode a candidate repressor of anthocyanin biosynthesis similar to PpMYB18.

### VcMYBC2 negatively regulates *VcMYBA1* and *VcUFGT* transcription

**Fig. 4:**
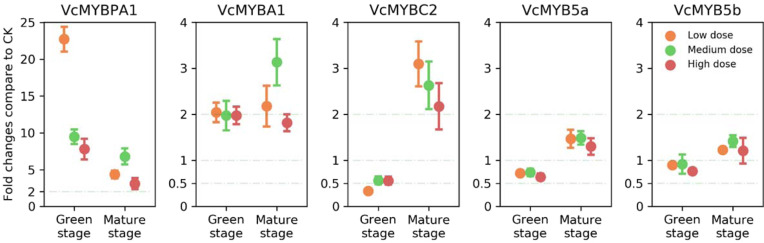
Expression analysis of anthocyanin biosynthesis-related MYB TFs. The values represent the fold-changes relative to the expression levels in the CK fruits (mean±s.e., *n* = 4)

Among the five identified MYB TF genes, the expression levels of *VcMYBPA1*, *VcMYBA1*, and *VcMYBC2* changed significantly following the UV-B treatment (Fig. 4). As a putative repressor, the *VcMYBC2* expression level was significantly downregulated in the green fruit stage, but was significantly up-regulated in the mature fruit stage, in response to UV-B irradiation. In terms of the anthocyanin biosynthesis activators, the *VcMYBA1* and *VcMYBPA1* expression levels were up-regulated by the UV-B treatment throughout the fruit development.

**Fig. 5:**
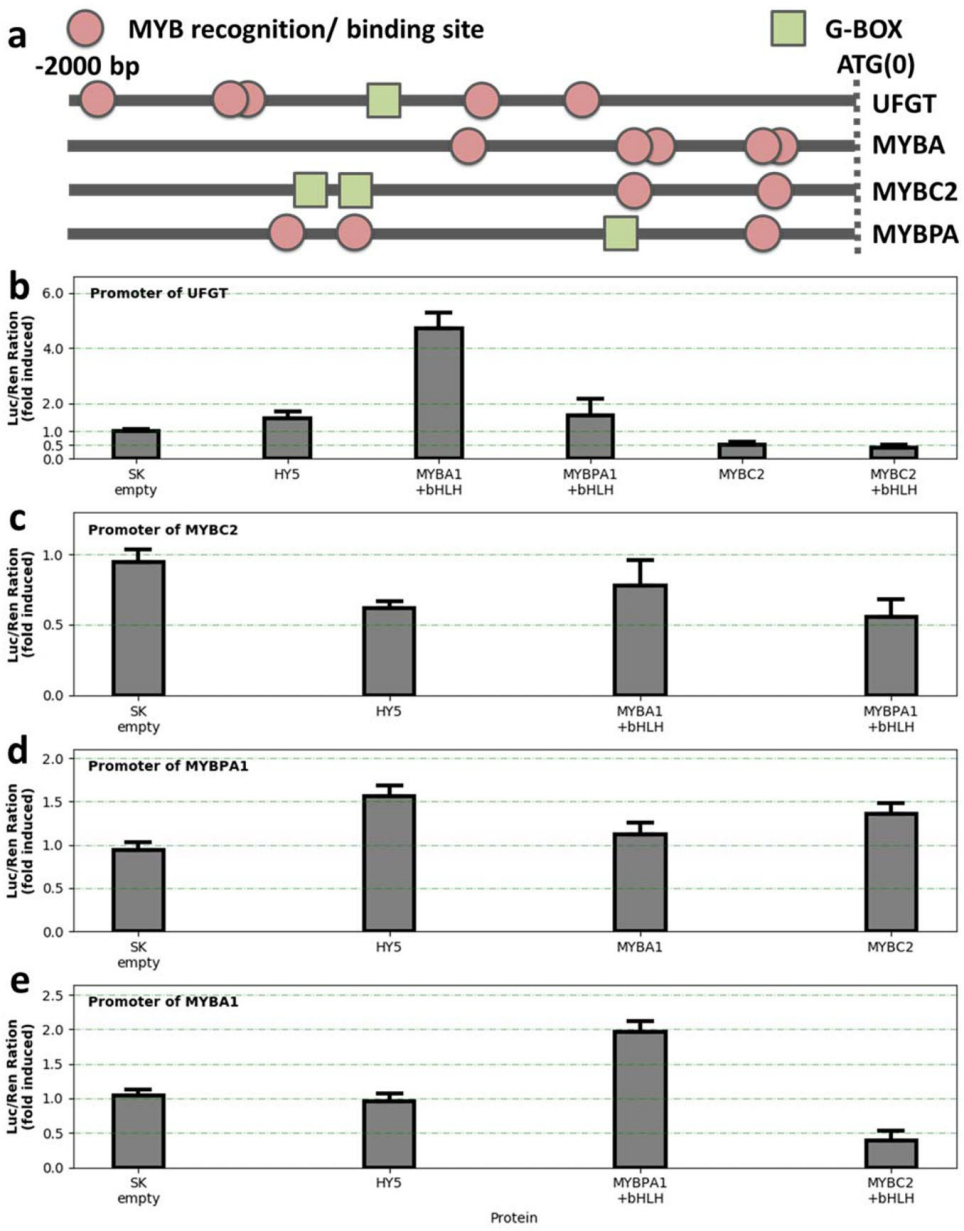
Dual-luciferace promoter assay of VcMYBs and VcUFGT by VcHY5 and VcMYBs transcription factors. **a** Schematic diagram of the predicted MYB-recognition/binding elements and G-box in the *VcUFGT*, *VcMYBA1*, *VcMYBPA1*, and *VcMYBC2* promoters. **b–e** Effects of VcHY5, VcMYBA1, VcMYBPA1, and VcMYBC2 on *VcUFGT* (**b**), *VcMYBC2* (**c**), *VcMYBPA1* (**d**), and *VcMYBA1* promoter activities (mean±s.e., *n* = 6)

Dual-luciferase assay on tobacco was conducted in this study, and the average Luc/Ren value of empty vector of Promoter-VcUFGT, VcMYBC2, VcMYBPA1 and VcMYBA1 were normalized to 1 in Fig. 5, with the raw value as 0.006655588, 0.006743612, 0.003020063 and 0.002384519, separately. The results suggested that the *VcUFGT* promoter can be activated by VcMYBA1, especiallywhen VcMYBA1 combines with bHLH TF (Fig. 5). Additionally, the *VcUFGT* promoter activity was repressed by VcMYBC2, with an average expression level that was only 39% of the control level. Thus, *VcMYBC2* is likely a repressor-type MYB TF gene. Additionally, compared to VcMYBC2 alone (value of Luc/Ren was 0.4937 relative to empty SK vector), VcMYBC2 repressed *VcUFGT* promoter activity greater when it was overexpressed with bHLH TF (Luc/Ren fold-induced value 0.3934). HY5-binding G-box elements were identified exclusively in the *VcMYBPA1*, *VcMYBC2*, and *VcUFGT* promoter regions (Fig. 5a), and consistent with this, HY5 activated the promoter activity of *VcMYBPA1* and *VcUFGT*, and repressed the *VcMYBC2* promoters (Fig. 5), but had no significant effect on the *VcMYBA1* promoter. Because many MYB-binding/recognition sites were detected in the promoters of three MYB TF genes (Fig. 5a), we hypothesized that there might be a regulatory interaction between the TFs. To test this hypothesis, we examined the interaction between these three MYB TFs and their gene promoters. The VcMYBPA1 activated *VcMYBA1* expression, whereas VcMYBC2 decreased *VcMYBA1* expression to 36% of that induced by the empty vector control. Additionally, VcMYBPA1 slightly downregulated the *VcMYBC2* expression level.

## Discussion

### Preharveat long-term UV-B treatment accelerated fruit coloration and ripening in blueberry

The anthocyanin concentration in the blueberry pericarp is mainly determined by the rate of biosynthesis and degradation (or transport)^31^. In the present study, anthocyanins were undetectable in the CK green fruits, but the UV-B treatments clearly increased the anthocyanin contents of all irradiated fruits. Additionally, the ratio of anthocyanins in UV-treated and initial pink fruits varied significantly (Fig. S1B). Therefore, we speculated that the anthocyanin accumulation mode in UV-B-treated green fruits differs from naturally occurred anthocyanin accumulation in mature fruits, which in turn suggesting that accelerated fruit coloration by UV-B exposure was caused primarily by the direct effects of UV-B on anthocyanin metabolism and not simply by the shift of ripening time. Unlike the light-induced anthocyanin accumulation in apples^32^ and pears^33^, the anthocyanin biosynthesis in blueberries is unaffected by an exposure to visible light or far-red light^34^. A previous analysis of short-term UV-B treatments suggested that compared with the effects of UV-A and UV-C rays, a preharvest UV-B irradiation can induce anthocyanin accumulation in blueberries^11^.

In another study, a postharvest short-term UV-B treatment increased anthocyanin biosynthesis within 3–6 h and transiently up-regulated the expression of the related structural genes, but there were no significant differences in the anthocyanin contents of the treated and control fruits after 24 h^10^. Our results indicate that a long-term preharvest UV-B treatment can increase the anthocyanin content of blueberries in the green and mature fruit stages. The anthocyanin content of the mature fruits treated with medium and high UV-B doses was 150% higher than that of the CK fruits, but the proportion of each anthocyanin component did not change significantly, suggesting that UV-B affects anthocyanin accumulation via changes to the upstream transcriptional regulation.

In apple^32^, pear^33^, and grape^35^, UFGT is considered to be a key enzyme for the rapid accumulation of anthocyanins following UV treatments. In blueberry, compared with the expression-level changes to the other structural genes, *VcUFGT* was initially unexpressed in green fruits (C_T_ value > 35 in qPCR assays), but its expression was considerably up-regulated by the UV-B treatment. The R2R3-MYB TFs in SG6 are the specific activators of anthocyanin biosynthesis. We determined that *VcMYBA1* regulates anthocyanin accumulation in blueberry by activating the transcription of *VcUFGT*. Moreover, we revealed the positive regulatory effects of *VcMYBPA1* on *VcMYBA1* expression in tobacco (Fig. 5e). This is consistent with the findings of an earlier study in which a low *VcMYBPA1* expression level resulted in the production of white blueberry mutant^36^.

### Identification of repressor-type MYBC2 that suppresses the expression of *MYBA1* and *UFGT* genes

In addition to MYB activators, MYB repressors also have regulatory functions related to anthocyanin biosynthesis in petunia^37,38^. and peach^19^. Günter et al. (2020) also assumed *VcMYBC2* may have repressor function on anthocyanin accumulation in blueberry. Here, our result further suggests that VcMYBC2 may function as a transcriptional repressor for *VcMYBA1* and *VcUFGT* via transient expression assay. To the best of our knowledge, this is the first study to verify the repressor function of *VcMYBC2*, while further experiments are required to be conducted to promote the understanding of this repressor in blueberry. During the fruit development, *VcMYBC2* was highly expressed in juvenile or green fruits, but the expression level significantly (*P* < 0.01) decreased as the fruit matured (Supplementary Fig. S2), which is consistent with the reported change in *VcMYBC2-L3* expression during the veraison period of grape^18^. In this study, the inhibitory effect of VcMYBC2 combined with a bHLH was greater than that of VcMYBC2 alone, implying that VcMYBC2 forms an MBW complex along with a bHLH TF to down-regulate *VcUFGT* transcription. Generally, MYB repressors can passively inhibit anthocyanin biosynthesis by competing with MYB activator to couple with bHLH protein in MBW complex, thus reducing its activation ability. In addition, R2R3 MYB repressor transforms the function of MBW complex from activation to repression through its repressor motif, which leads to active inhibition of downstream gene transcription. In response to UV-B exposure, the expression of MYB repressor genes, such as *VvMYBC2* and *VvMYB4*, reportedly decreases to promote the accumulation of flavonoid secondary metabolites^18^. Similarly, *VcMYBC2* expression was downregulated by UV-B radiation at the green fruit stage in this study, which promotes anthocyanin accumulation at the green fruit stage. However, *VcMYBC2* expression increased in mature fruits exposed to UV-B light, which prevents the overaccumulation of anthocyanins during the mature fruit stage. In grapes, the overexpression of the anthocyanin activator gene *VvMYBA1* and the PA activator genes *VvMYBPA1* and *VvMYBPA2* can induce the expression of *VvMYBC2-L1* in hairy roots^18,39,40^. A similar negative feedback loop in peach prevents cells from accumulating excessive amounts of anthocyanins and PAs. In fact, co-expression of VcMYBC2 with the activators, MYBA1 and MYBPA1, was detected in previous research^21^. However, our dual-luciferase assay results were inconsistent with an induction of *VcMYBC2* expression by *VcMYBA1* and *VcMYBPA1*. Thus, there may be other regulatory networks in blueberries that regulate *VcMYBC2* expression to avoid excess amounts of anthocyanins in the mature fruit.

**Fig. 6:**
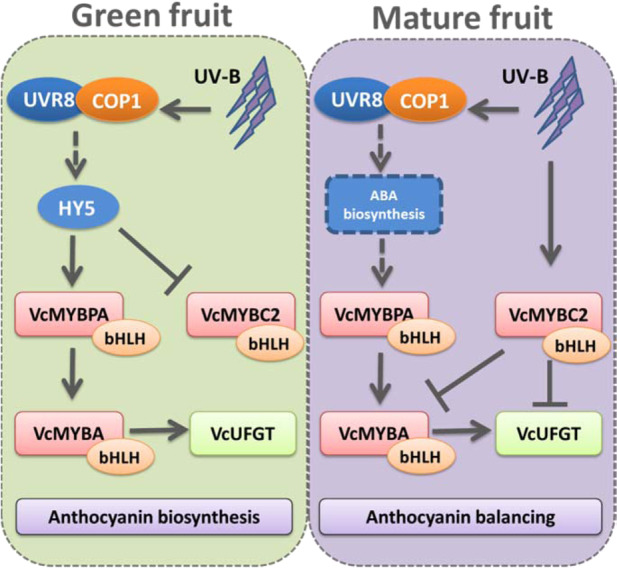
Proposed model of the stage-specific modification of UV-B on anthocyanin biosynthesis pathway in green and mature blueberry fruits. Arrow with solid line represents the regulation relationship which has been verified by dual-luciferase assay in this study, the dotted arrow represents the regulation relationship which has been proved in other reference articles

### UV-B modifies blueberry anthocyanin metabolism in a stage-dependent manner

We propose the model of possible molecular mechanisms underlying UV-B effects on fruit coloration and ripening (Fig. 6). In green fruit stage, a UV-B treatment promotes the expression of *VcHY5*, which activates *VcMYBPA1* and *VcMYBA1* transcription. In *A. thaliana*, HY5 induces the expression of key genes related to pigment accumulation through the T/G-box motif in the promoter of structural genes or TFs involved in flavonoid metabolism^22^. Recently, HY5 protein has been proven that it cannot activate transcription alone, while it forms a complex with specific TFs to modulate transcription in plants^34^. Our gene expression data revealed slight (fold-change < 2) but significant changes in the transient expression of *VcMYBPA1*, *VcMYBC2*, and *VcUFGT* are induced by HY5. The expression of *VcMYBA1*, which lacks a G-box in its promoter, was unaffected by VcHY5. In green blueberry fruits, the UV-B induced expression of *HY5* and promoted flavonoid biosynthesis, which is consistent with the results of earlier investigations on apple^8^, pear^32^, and grape^41^. The up-regulated activator-type MYBs enhanced the expression levels of anthocyanin biosynthesis-related structural enzymes, such as *VcUFGT* and *VcF3'5’H*. The activated VcHY5 also inhibits the expression of *VcMYBC2* to minimize the repression of *VcMYBA1* and *VcUFGT* expression, thereby promoting anthocyanin accumulation.

However, there was no significant change in *VcHY5* expression when the blueberries were exposed to UV-B light during the ripening period, even though the expression levels of some *MYB*s related to anthocyanin synthesis increased significantly. Earlier investigations indicated that UV-B irradiation can increase abscisic acid (ABA) biosynthesis in plant organs^42,43^, and increased *COP1* levels can promote plant responses to ABA by promoting the binding of ABSCISIC ACID INSENSITIVE (ABI) to their target promoters^44^. We thus speculate that the increased *COP1* levels in response to UV-B activated ABA response pathway, which primarily caused the up-regulated *VcMYBPA1* expression in mature fruits^42–44^. Furthermore, the up-regulated expression of *VcMYBC2* in response to UV-B through unknown pathways, inhibits anthocyanin accumulation via the inhibition of *VcMYBA1* and *VcUFGT* to balance the anthocyanin content.

### Possible utility of a preharvest UV-B exposure to regulate blueberry fruit development

Fresh blueberries are favored by consumers because they are nutrient-rich fruits with a unique flavor, but they have a short shelf-life. Moreover, blueberry fruit clusters ripen relatively inconsistently, which can considerably increase the costs associated with artificial harvesting^3^. Therefore, improving the regulation of the ripening period is very important for ensuring the stable commercial production of blueberry fruits.

We demonstrated the possibility of applying artificial UV-B with environment-relevant dose in regulating tree-fruit production. Compared with fruits exposed to short-term UV by Yang et al.^11^, long-term UV supplementation induced more anthocyanin accumulation than short-term treatment. In this study of blueberry fruits, even a low UV-B dose accelerated fruit ripening and increased the secondary metabolite contents, implying that blueberry fruits on shrubs are very sensitive to UV-B irradiation. In the 1980s, researchers used UV lamps to treat blueberries with a UV-B dose equivalent to that of natural UV-B light from the sun, which substantially reduced the fruit size and the formation of wax on the fruit surface^45^. In the current study, we exposed blueberry fruits to biologically effective UV-B doses, which did not damage the fruits, even when the UV-B dose was 30% higher than that on an average summer day. These findings confirm the importance of applying biologically effective UV-B doses for cultivating fruits in a greenhouse.

Ultraviolet-B can inhibit the growth and development of plant leaves and roots via regulating auxin homeostasis, including photooxidative damage, biosynthesis, binding, and/or degradation^46^. We also found similar phenomena on blueberry leaves during the treatment. Blueberries are non-climatic fruits, and the fruit development and maturity can be regulated by auxin and abscisic acid. The biosynthesis of these two hormones is generally affected by UV-B^42–44,46^. For blueberry fruits, only massive UV-B exposure can cause this inhibitory effect^46^. In comparison, environment-relevant doses of ultraviolet can promote the development and maturity of the fruit. Henry-Kirk et al.^8^ also found that solar UV-B can promote the development of apple fruits through the comparison between fruits with and without solar UV-B dose. Compared with leaves and roots, blueberry and apple fruits have thick and opaque peels that can accumulate anthocyanins, which can effectively reduce the inhibitory effect of UV-B on pulp development, while the promotion of UV-B on pulp development may be caused by the hormone regulatory network in the different organs. These organ-specific responses still need further study and it is a potential cut-in point in attempts to transform UV-B from harmful environmental factors to beneficial technologies for fruit trees. We also detected an increase in the sugar accumulation of blueberry fruits treated with a low UV-B dose. A low UV-B dose reportedly promotes the sugar accumulation in peach fruits by increasing the expression of sugar transporter genes^47^. In addition to accelerating fruit development, an exposure to UV-B light can also increase the uniformity of berry ripening in one cluster, which highlights the potential utility of UV-B treatments for regulating the ripening of fruit clusters. These dose-dependent changes in fruit development and quality indicate that appropriate UV-B doses will need to be determined to improve agronomic practices to increase fruit production and quality. Our discovery on accelerated fruit size development, increased sugar contents, and more uniform berry ripening in cluster induced by UV-B exposure, would be of great interest for future work.

## Conclusion

The results of this study indicate that a supplementary UV-B treatment can significantly influence the ripening and nutritional quality of blueberry, which is one of the most important fruit crops worldwide. Because of the popularity of cultivating fruits in greenhouses, an artificial UV-B treatment may be useful for improving crop physiology to produce fruits with enhanced nutritional properties. We also determined that a specific regulatory network involving *VcMYBA1*, *VcMYBPA1*, and *VcMYBC2* mediates UV-B-induced anthocyanin accumulation. This network may form the theoretical basis for further enhancements of blueberry fruit quality. The data presented herein proposed that blueberry fruits exposed to medium or high UV-B doses ripen faster and more consistently than untreated fruits, with no detrimental effects on fruit quality. However, the practical utility of UV-B irradiations for blueberry production will need to be verified.

## Supplementary information

Supplemental material
