# Supplementary material for: Preharvest long-term exposure to UV-B radiation promotes fruit ripening and modifies stage-specific anthocyanin metabolism in highbush blueberry
Source: Hortic Res. 2021 Apr 1;8:67. doi: 10.1038/s41438-021-00503-4 (PMC8012384; doi:10.1038/s41438-021-00503-4)

Supplementary Table S1. Gene-specific primers used for qRT-PCR.

| Gene | Accession number | Forward primer (5' to 3') | Reverse primer (5' to 3') | Source |
| --- | --- | --- | --- | --- |
| VcGAPDH | AY123769.1 | GGTTATCAATGATAGGTTTGGCA | CAGTCCTTGCTTGATGGACC | Reference 13 |
| VcMYBA1 | CUFF.51383.1 | AAAGGGTGCTTGGACTGAGG | CTGCCCTGTAAGGAACTTGGT | Designed by this study |
| VcMYBPA1 | CUFF.51789 | CCACCAAAGAAGAGGAGGAC | CCATTGCCATCGAATTTAGAC | Reference 13 |
| VcMYBC2 | CUFF.47076.1 | GGGAGCTCGAAACCATCGAA | TGATCGGCTTTCCATGACCG | Designed by this study |
| VcMYB5a | CUFF.52595.1 | GTGTTGCAGCAAAGTGGGAC | CGGAGTCGGCAACTCTTACC | Designed by this study |
| VcMYB5b | CUFF.43191.1 | AGTCTCGAGCCACAAGCAAA | TCGGCTTTGATATTGGCGGT | Designed by this study |
| VcPAL | AY123770.1 | TTACAACAATGGGTTGCCCT | CCTGGTTGTGTGTCAGCACT | Reference 13 |
| VcCHS | JN654702.1 | CTTGACTGAGGAAATCTTGAAGG | AGCCTCTTTGCCCAATTTG | Reference 13 |
| VcF3H | AB610765.1 | GGGTTTTCCAGGTCGTCGAT | TATCGAACCGCAGCTTCTCC | Reference 13 |
| VcF3'H | AB694901.1 | CGAGATTCGATGCGTTTCTGAGTG | GATTTCGGTATCGGTGAGCTTCC | Reference 13 |
| VcF3'5'H | KP218506.1 | GCCTATGCATAGCAAGTGGGA | TTGATTCGGTGCCTTGAGAAT | Reference 13 |
| VcDFR | KF960989.1 | CACTGAGTTTAAGGGGATTCCTAAGG | CCCTTCTCCCTACAAGTGTCAATGG | Reference 13 |
| VcANS | JN654701.1 | CTTCATCCTCCACAACATGGT | GCTCTTGTACTTCCCATTGCTC | Reference 13 |
| VcUFGT | CUFF.20951 | AGTTTGCTTTGAAGGCTGTTG | ATGTGCTGGTGTGCATTTG | Reference 13 |
| VcUVR8 | CUFF.6010.1 | TGGTTATGGTGGCATGTGGAT | AACCTATGAGGCACAAGGTGG | Designed by this study |
| VcHY5 | CUFF.47089.1 | GAGAGTACCGGAGATCGGC | TTTATCGGCCGGACTTCTGC | Designed by this study |
| VcCOP1-1 | CUFF.28774.1 | CGAACGCATGGCCTTCTTTG | GAATTGCCTGCGTTCATCCG | Designed by this study |
| VcCOP1-2 | CUFF.8155.1 | GCTCAGCAGACCACCATATCC | CCACTGAACACATGGAGTGGA | Designed by this study |

Supplementary Table S2. Gene-specific primers used for dual-luciferase assay

| Vector gene | Sequence 5’-3’ | |
| --- | --- | --- |
| SK_VcMYBA1 | Forward primer | GGCGGCCGCTCTAGAACTAGTATGATTCACTTAAAGGGTGCTA |
|  | Reverse primer | GATAAGCTTGATATCGAATTCTCACCGTTATCCACCATCATGG |
| SK_VcMYBC2 | Forward primer | GGCGGCCGCTCTAGAACTAGTATGAGGAAGCCATGTTGTGAGA |
|  | Reverse primer | GATAAGCTTGATATCGAATTCTCATCTAAAGAGAGGAAGGGTG |
| SK_VcMYBPA1 | Forward primer | GGCGGCCGCTCTAGAACTAGTATGAATTATCTGAGACCAGATA |
|  | Reverse primer | GATAAGCTTGATATCGAATTCTCAAATCAACAATGATTCGGCA |
| SK_VcHY5 | Forward primer | GGCGGCCGCTCTAGAACTAGTATGGAATTAATGAATTTTGAAG |
|  | Reverse primer | GATAAGCTTGATATCGAATTCTTACTTCCTCCCCTCTTGCATG |
| SK_MdbHLH | Forward primer | GGCGGCCGCTCTAGAACTAGTATGGCTGCACCGCCGCCAAGCA |
|  | Reverse primer | GATAAGCTTGATATCGAATTCTTAAGAGTCAGATTGGGGTATA |
| LUC_MYBC2 | Forward primer | TCCACTAGTTCTAGAGCGGCCGCAAAGTAACTAGTACATTCTCTA |
|  | Reverse primer | TGTTTTTGGCGTCTTCCATGGTCTACTCACACAGAGTACATAC |
| LUC_MYBPA1 | Forward primer | TCCACTAGTTCTAGAGCGGCCGCTGTTTTTACTCGTTGCGTTTTGACC |
|  | Reverse primer | TGTTTTTGGCGTCTTCCATGGGCAACTTAATAAGCACGTGGAG |
| LUC_UFGT | Forward primer | TCCACTAGTTCTAGAGCGGCCGCGGGGTCAAGAATCTGTAGTATA |
|  | Reverse primer | TGTTTTTGGCGTCTTCCATGGGGTTATATTTTTGGTGGTGGGC |
| LUC_MYBA1 | Forward primer | TCCACTAGTTCTAGAGCGGCCGCTGGTCTCGGTTTACATAAACAG |
|  | Reverse primer | TGTTTTTGGCGTCTTCCATGGCCATCTCAGCCTACAGCTTTTT |


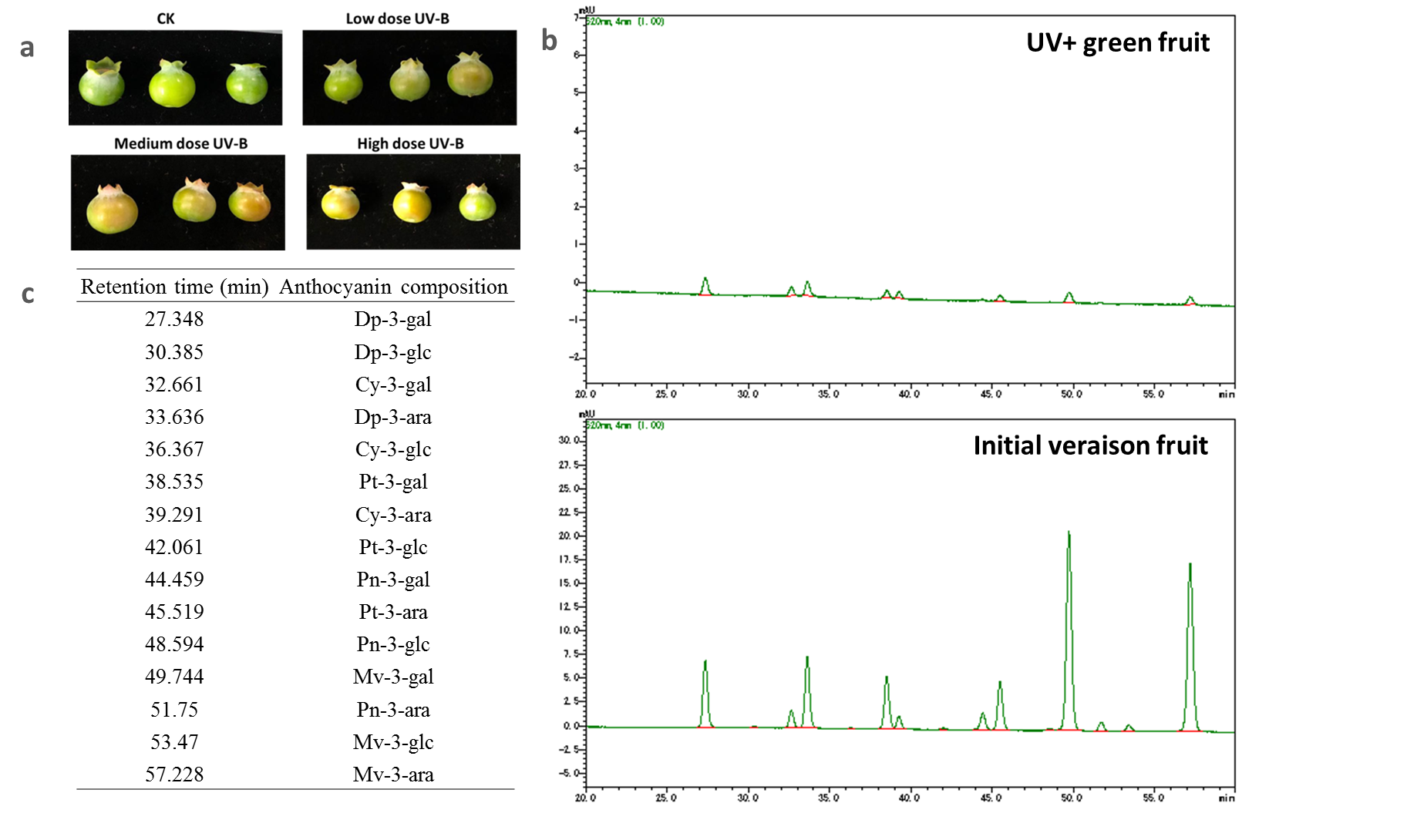


Supplementary Figure S1. Anthocyanin accumulation in green fruits after 10-day UV-B treatment, a; Chromatogram of anthocyanins in ‘O’Neal blueberry fruits, b; Peak annotation of anthocyanin chromatogram; c.

Supplementary Figure S2 Relative expression levels of *VcMYBs* in blueberry fruits at green or ripening stage (mean ±s.e., n=4)


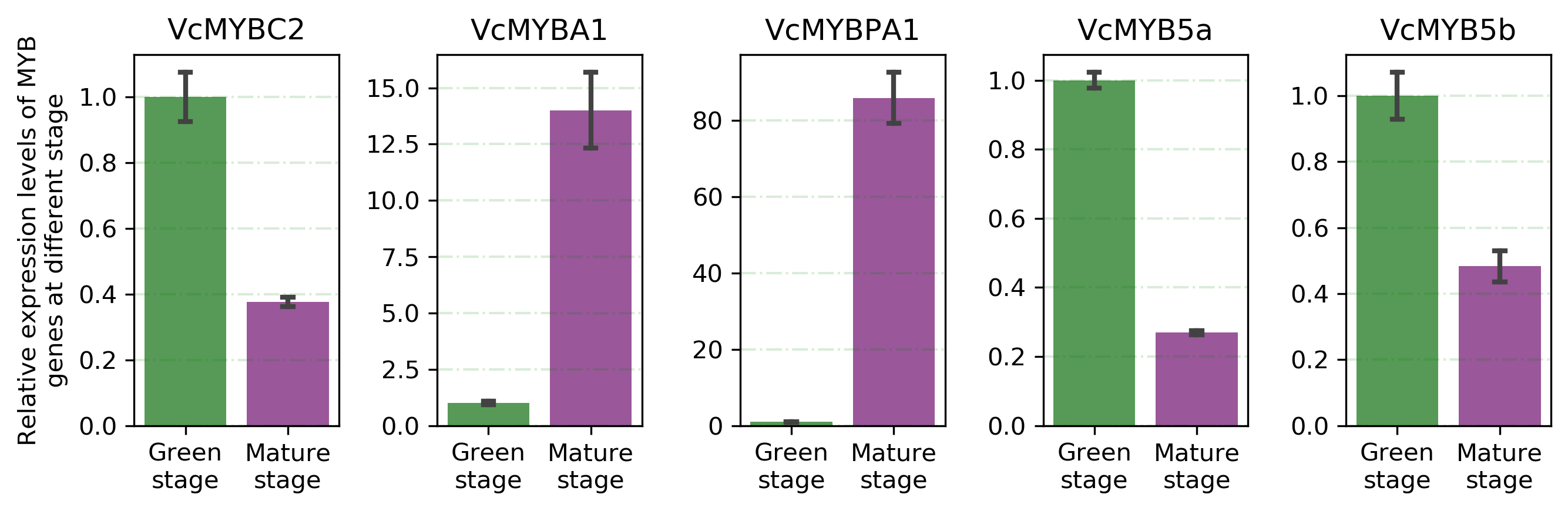

Supplement: Supplementary file 1 — Supplemental material [file 41438_2021_503_MOESM1_ESM.docx]
